# Nicotine's Defensive Function in Nature

**DOI:** 10.1371/journal.pbio.0020217

**Published:** 2004-08-17

**Authors:** Anke Steppuhn, Klaus Gase, Bernd Krock, Rayko Halitschke, Ian T Baldwin

**Affiliations:** **1**Department of Molecular Ecology, Max Planck Institute for Chemical EcologyJenaGermany

## Abstract

Plants produce metabolites that directly decrease herbivore performance, and as a consequence, herbivores are selected for resistance to these metabolites. To determine whether these metabolites actually function as defenses requires measuring the performance of plants that are altered only in the production of a certain metabolite. To date, the defensive value of most plant resistance traits has not been demonstrated in nature. We transformed native tobacco*(Nicotiana attenuata)* with a consensus fragment of its two *putrescine N-methyl transferase (pmt)* genes in either antisense or inverted-repeat (IR*pmt*) orientations. Only the latter reduced (by greater than 95%) constitutive and inducible nicotine. With D_4_-nicotinic acid (NA), we demonstrate that silencing *pmt* inhibits nicotine production, while the excess NA dimerizes to form anatabine. Larvae of the nicotine-adapted herbivore Manduca sexta (tobacco hornworm) grew faster and, like the beetle *Diabrotica undecimpunctata,* preferred IR*pmt* plants in choice tests. When planted in their native habitat, IR*pmt* plants were attacked more frequently and, compared to wild-type plants, lost 3-fold more leaf area from a variety of native herbivores, of which the beet armyworm, *Spodoptera exigua,* and *Trimerotropis* spp. grasshoppers caused the most damage. These results provide strong evidence that nicotine functions as an efficient defense in nature and highlights the value of transgenic techniques for ecological research.

## Abstract 2

Transgenic plants confirm the role that nicotine has in protecting tobacco plants from predators in nature and demonstrates the power of transgenic tools in studying ecological interactions in the field

## Introduction

Plants produce many secondary metabolites, of which some are thought to function as direct defenses against pathogens and herbivores by reducing their performance, survival, and reproduction. Numerous plant allelochemicals with antiherbivore properties are classified according to their mode of action (e.g., toxins, antifeedants, antidigestive proteins, etc.) (Bennett and Wallsgrove 1994) and have been used in agriculture to control insect pests (Hedin 1991). The fact that a secondary metabolite reduces herbivore performance does not by itself demonstrate that the endogenously expressed metabolite functions defensively in the plant's natural environment (Bell 1987), because the evolutionary interaction between herbivores and their host plants may have reduced the defensive efficacy of the metabolite. Phytophagous insects have evolved various strategies to cope with allelochemicals (Karban and Agrawal 2002) and tend to tolerate, or even co-opt, plant defenses for their own defenses (Wink and Theile 2002).

Pharmacological studies demonstrating a resistance effect of metabolites applied to plants or artificial diets (Yamamoto et al. 1968; Bowers and Puttick 1988), and studies using heterologously expressed genes in agricultural systems (Carozzi and Koziel 1997; Hilder and Boulter 1999), represent a first step in evaluating the defensive function of a secondary metabolite. The interpretation of these studies is confounded by both the altered ecological context in which the resistance is measured and the altered chemical milieu, which is also known to influence the defensive function of a metabolite. Stronger evidence for resistance effects of allelochemicals arises from studies establishing correlations between plant resistance against herbivores and the genetically variable accumulation of secondary metabolites (Berenbaum et al. 1986; Shonle and Bergelson 2000) or from studies demonstrating the defensive role played by a suite of elicited metabolites (Orozco-Cardenas et al. 1993; Baldwin 1998; Halitschke and Baldwin 2003). Ideally, the benefits of a putative defense trait should be determined in plants differing only in a single gene that controls the expression of a resistance trait and are otherwise identical (Bergelson and Purrington 1996). To date, studies measuring resistance of “near isogenic” lines with altered metabolite accumulations (Jackson et al. 2002) provide the strongest evidence for their resistance, but these lines, which are created by repetitive backcrossing, are likely to differ in many loci linked to the target locus, which may also affect resistance. Such problems of genetic linkage have been overcome through the use of genetic transformation to explore the fitness effects of herbicide resistance (Bergelson et al. 1996; Purrington and Bergelson 1997) and pathogen resistance (Tian et al. 2003) in field populations of *Arabidopsis*. In this study, we use transgenic silencing to alter a single putative resistance trait—the production of nicotine—and thereby establish its contribution to plant resistance in the field.

The pyridine alkaloid nicotine is one of the best-studied putative plant resistance traits. Because it can interact with the acetylcholine receptors in the nervous systems of animals, nicotine is extremely toxic to most herbivores and, consequently, was one of the first insecticides used to control pests in agriculture (Schmeltz 1971). Evidence for the resistance value of nicotine arises from the agricultural practice of using nicotine sprays and genotypes of cultivated tobacco differing in nicotine levels (Jackson et al. 2002). Although nicotine is widely toxic, insects adapted to nicotine-producing plants have evolved resistance to this alkaloid (Glendinning 2002). The tobacco specialist Manduca sexta (tobacco hornworm) tolerates doses of nicotine that are fatal to unadapted herbivores but grows more slowly on high-nicotine diets (Appel and Martin 1992; Wink and Theile 2002). Other studies suggest that M. sexta might even be better defended by dietary nicotine against its parasitoid, *Cotesia congregata,* which suffers higher mortality when parasitizing larvae fed on high- rather than low-nicotine diets (Barbosa et al. 1986; Thorpe and Barbosa 1986). Thus, the coevolutionary arms race between nicotine-producing plants and their adapted herbivores may have reduced the defensive value of nicotine.

In the native tobacco species Nicotiana attenuata and *N. sylvestris,* nicotine is the most abundant alkaloid. Elicitation of N. attenuata with jasmonic acid methyl ester (MeJA) in its native habitat increases nicotine content, which is correlated with enhanced plant fitness when plants are attacked (Baldwin 1998). However, herbivore attack and MeJA elicitation (as well as the plant's endogenous jasmonic acid cascade [Halitschke and Baldwin 2003]) regulate many resistance traits, including trypsin protease inhibitors (TPIs), diterpene glycosides, and volatile emissions involved in indirect defense. Hence, nicotine is only one of a suite of putative defense traits elicited by herbivore attack, and its specific role remains to be determined.

In laboratory trials, resistance benefits of nicotine production against M. sexta larvae were established using transgenic N. sylvestris plants silenced in their nicotine biosynthesis by antisense expression of putrescine N-methyl transferase (PMT). Plant consumption and the performance of M. sexta larvae were negatively correlated with constitutive nicotine levels in laboratory feeding trials (Voelckel et al. 2001); whether this result applies to plants in their natural habitat is unclear. To examine the resistance effect of nicotine, we transformed N. attenuata with inverted-repeat *pmt* (IR*pmt*) and antisense *pmt* constructs and found that only IR*pmt* plants had strongly reduced nicotine content. We characterized the defense and growth phenotypes of two independently transformed homozygous IR*pmt* lines and found that measured direct and indirect defenses did not differ from those of the wild-type (WT) plants, except for a dramatic reduction (greater than 95%) of MeJA-elicited and constitutive nicotine production and an increase in anatabine content. In pulse-chase experiments with D_4_-nicotinic acid (NA) ethyl ester, we demonstrated that the increased anatabine likely results from a dimerization of the NA that would normally have been used in nicotine biosynthesis. In feeding trials, M. sexta larvae preferred and grew faster on IR*pmt* than WT leaves. We transplanted WT and IR*pmt* plants into N. attenuata's native habitat in southwestern Utah and elicited a subset with MeJA. Several naturally occurring herbivore species attacked and damaged unelicited IR*pmt* plants more than unelicited or elicited WT and elicited IR*pmt* plants. These results demonstrate that nicotine functions as an effective resistance trait under natural conditions.

## Results/Discussion

### IR*pmt* Constructs Silence Nicotine Production

**Figure 1:**
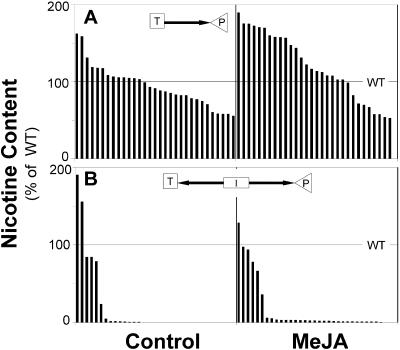
Comparison of Antisense and Inverted-Repeat Silencing of *pmt* Nicotine content (mean of 5–6 plants/line) normalized to mean of WT of unelicited (control) N. attenuata plants and plants 5 d after elicitation with 150 μg of MeJA per plant from independent lines transformed with (A) antisense *pmt* constructs and (B) an IR*pmt* construct. In contrast to the 31 lines transformed with the antisense *pmt* construct, 29 of the 34 IR*pmt* lines had dramatically reduced constitutive and MeJA-induced nicotine levels. T, terminator; P, promoter; I, spliceable intron; arrow, 950-bp consensus fragment of *pmt1* and *pmt2*. For details of transformation constructs see Protocol S1.

Nicotine accumulation was not reduced in most of the independent lines transformed with antisense *pmt* constructs (25 lines of pNATPMT1 and six lines of pCAMPMT1) compared to WT (Figure 1A). None of the five lines with lower nicotine accumulation in the T_1_ screen had nicotine levels lower than those of WT in the homozygous T_2_ generation. In contrast, 29 of 34 independently transformed lines with the IR*pmt* construct pRESC5PMT had dramatically reduced constitutive and MeJA-induced nicotine accumulations (Figure 1B). The suppression of nicotine accumulation was stable during plant development and when plants were grown in the glasshouse or in the field in Utah. Clearly, inverted-repeat constructs are more efficient at silencing the expression of endogenous genes, as has been previously described (Wesley et al. 2001).

### Genomic and Transcriptional Characterization

Two homozygous T_2_ IR*pmt* lines (108 and 145) with reduced nicotine levels were further characterized. Southern blot analysis using a probe hybridizing to the selective marker in the IR*pmt* construct demonstrated that both lines contained a single insertion (Figure S1). Transformation with a pRESC transformation vector allowed the transferred DNA (T-DNA) and flanking DNA at the insertion site to be recovered from the plant genomic DNA. These experiments demonstrated that the T-DNA integrated into the N. attenuata genome at a single site in each line, since all sequenced clones from a line (108, *n* = 4; 145, *n* = 5) contained the same flanking sequence (see Figure S1 and Protocol S1).

**Figure 2:**
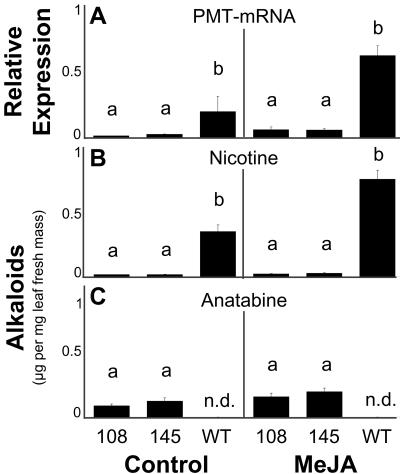
PMT Transcript and Alkaloid Levels of IR*pmt* Lines Mean (± SE) relative PMT mRNA transcript levels in the roots (A), and leaf levels of (B) nicotine and (C) anatabine, in two independent lines of IR*pmt*-transformed (108 and 145) and WT N. attenuata plants. Elicited (150 μg of MeJA) and unelicited (control) plants were harvested at 10 h for transcript (A) and at 4 d for alkaloid (B and C) quantification. Both IR*pmt* lines had significantly reduced PMT transcript and nicotine but featured anatabine not present in WT plants. Lowercase letters signify differences at *p* ≤ 0.01, Bonferroni corrected ([A] *n* = 3, ANOVA: F_2,12_ = 12.55; [B] *n* = 8–10, ANOVA: F_2,50_ = 135.4; [C] *n* = 8–10, ANOVA: F_2,50_ = 39.611]. n.d., not detected.

Transcripts of the *pmt* genes in the two lines were significantly reduced to approximately 10% of the constitutive and MeJA-induced WT mRNA levels (Figure 2A), demonstrating that the targeted genes were successfully silenced.

### Metabolic Consequences of *pmt* Silencing in N. attenuata

Consistent with the observed silencing of *pmt* transcripts, the constitutive and induced nicotine levels in transformed plants of both lines were dramatically reduced to 3%–4% of the levels found in WT plants (Figure 2B). All 29 IR*pmt* lines with reduced nicotine levels accumulated the alkaloid anatabine, which was not detected in WT plants. Constitutive and MeJA-induced total (nicotine, anabasine, and anatabine) alkaloid contents of the two IR*pmt* lines were about one-half and one-third of the WT levels, respectively, of which anatabine comprised 30% and 23% (Figure 2C). Levels of anabasine representing 20% of the constitutive and 8% of the MeJA-elicited total alkaloid contents in WT plants were unchanged in IR*pmt* plants (Figure S2). Elevated anatabine levels were also found in recently published studies with antisense *pmt* transformation of *N. tabacum;* elevated anatabine levels did not affect transcript levels of other genes encoding enzymes involved in alkaloid metabolism (Chintapakorn and Hamill 2003).

**Figure 3:**
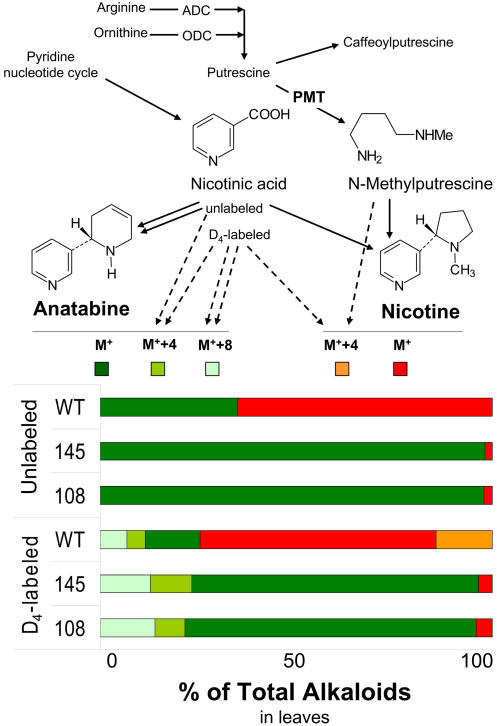
Alkaloid Biosynthesis and the Consequences of a NA Oversupply Biosynthesis scheme and proportion of unlabeled (M^+^) and labeled (M^+^+4, M^+^+8) nicotine and anatabine in the leaves of two independently transformed N. attenuata IR*pmt* lines (108 and 145) and WT plants 5 d after elicitation with 150 μg of MeJA per plant. Plants were grown in hydroponic solutions and supplied with either unlabeled or D_4_-ring-labeled NA ethyl ester (1 mM) 24 h after elicitation (*n* = 3 or 4). The oversupply of NA resulted in the formation of anatabine even in WT plants from both labeled exogenous and unlabeled endogenous NA pools.

Anatabine consists of a pyridine and a piperideine ring. Both are likely derived from NA, which is also the precursor of the pyridine ring of nicotine (Leete and Slattery 1976). Disrupting nicotine biosynthesis at the formation of the pyrrolidine ring by silencing PMT activity might cause an oversupply of the NA used in the biosynthesis of anatabine. Feeding the roots of hydroponically grown MeJA-elicited WT plants with NA ethyl ester resulted in formation of anatabine at levels of about a third of the total alkaloids (nicotine and anatabine) (Figure 3); in the IR*pmt* lines, anatabine constitutes 98% of the total alkaloids. Feeding plants with D_4_-NA ethyl ester results in the formation not only of D_4_-nicotine and D_4_-anatabine but also of D_8_-anatabine, demonstrating that the last integrates two D_4_-NA units. When these experiments are conducted with WT plants, about half of the anatabine is labeled, suggesting that the unlabeled half was formed from endogenous unlabeled NA. In addition, about one-fourth of the WT nicotine was D_4_-nicotine. In IR*pmt* plants, in contrast, only traces of D_4_-nicotine were found, but one-third of the anatabine was either D_4_- or D_8_-labeled. In summary, exogenously supplied NA is taken up by the roots of N. attenuata plants and used in alkaloid biosynthesis, and an oversupply of NA results in the formation of anatabine. These results support the hypothesis that the silencing of *pmt* disrupts nicotine biosynthesis, causing an oversupply of NA and the subsequent formation of anatabine.

IR*pmt* plants did not differ from WT plants in any other measured secondary metabolite or growth parameter. Constitutive or MeJA-induced levels of caffeoylputrescine, chlorogenic aid, rutin (Figure S2), TPI activity, or the release of *cis*-α-bergamotene (Figure S3) in IR*pmt*-transformed plants did not differ from those of WT plants. Rosette-stage and elongation-stage growth in individual pots in both the glasshouse and the field (Figure S4) did not differ between WT and IR*pmt* lines, and transformed lines were not visually or morphologically distinguishable from WT plants. Hence, the IR*pmt* plants represent an ideal construct with which to examine the ecological consequences of nicotine production.

### Effects of Nicotine Silencing on N. attenuata Herbivores

M. sexta larvae reared on IR*pmt* plants in the glasshouse gained significantly more mass and changed instars faster than larvae reared on WT plants (*n* = 17–20; ANOVA: *p* < 0.01, *p*_WT-PMT108_ < 0.02, *p*_WT-PMT145_ < 0.01). The differences were comparable to those observed for M. sexta larvae reared on nicotine-enriched artificial diets (Parr and Thurston 1972; Appel and Martin 1992) or on nicotine-enhanced WT (Baldwin 1988) or antisense-*pmt*–transformed N. sylvestris plants (Voelckel et al. 2001). Two-thirds of freshly eclosed M. sexta larvae, given the choice between leaf material from WT or IR*pmt* (108) plants, preferred to initiate feeding on the latter (*n* = 43; Chi^2^ = 6.7, *p* < 0.01). Such behavior suggests that nicotine plays an important role in determining feeding sites of M. sexta larvae*,* as has been suggested in a study with cultivated tobacco (Kester et al. 2002). While the relative toxic effects of anatabine and nicotine remain unstudied, these results are likely to underestimate the influence of nicotine on M. sexta choice and performance, because IR*pmt* plants had enhanced levels of anatabine.

**Figure 4:**
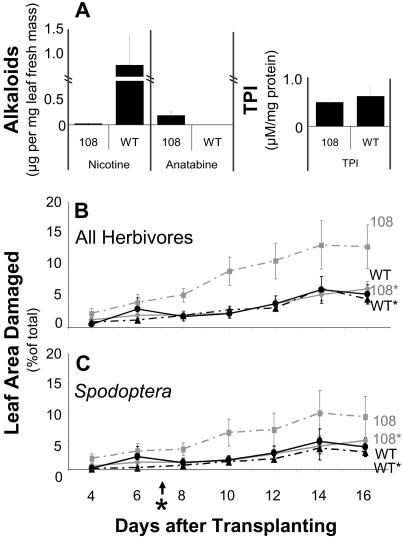
Herbivore Damage to IR*pmt* and WT N. attenuata Plants in Nature (A) Leaf alkaloids (nicotine and anatabine) and TPIs 7 wk after transplantation (*n* = 6). Mean (± SE) percentage total leaf area damaged by (B) all herbivores and (C) only by Spodoptera exigua on WT N. attenuata plants and plants transformed with an IR*pmt* construct (108) that were either untreated (solid lines) or elicited (dotted lines; asterisk) with MeJA 7 d after plants were transplanted into a field plot in a native habitat. Differences between 108 and WT, 108*, and WT* are significant at *p* ≤ 0.05 (*n*_PMT_ = 36, *n*_WT_ = 50, *n*_PMT*_ = 28, *n*_WT*_ = 27; [B] ANOVA: F_3,822_ = 5.73, *p* = 0.001; [C] ANOVA: F_3,822_ = 4.6, *p* = 0.004). Plants of the nicotine-deficient transformed line 108 suffered significantly higher leaf area damage than did WT plants, but when line 108 was elicited, leaf damage by all herbivores was reduced to WT levels.

Since secondary metabolism is known to be sensitive to environmental parameters that differ between glasshouse and field conditions (e.g., UV-B influence; Caldwell et al. 1983), nicotine, anatabine, and TPI levels of WT and IR*pmt* plants grown in the field plantation were analyzed: they were found not to differ from plants grown under laboratory conditions (Figure 4A). A M. sexta feeding choice test evaluating the larvae's choice between field-grown WT and IR*pmt* plants (*n* = 57; Chi^2^ = 7.74, *p* < 0.01) verified the results described above for the same experiment conducted with glasshouse-grown plants. Thus, the phenotype of glasshouse-grown IR*pmt* plants was not altered by growth under field conditions. In addition, choice tests with field-collected *D. undecimpunctata,* which was observed colonizing only IR*pmt* plants in the field plantation, revealed that 77% of these beetles preferred the nicotine-deficient IR*pmt* leaf material over WT (*n* = 35; Chi^2^ = 10.31, *p* < 0.001). Another beetle species observed occasionally on WT plants, *Trichobarus mucorea,* does not distinguish between WT and IR*pmt* leaf material in choice tests (*n* = 19; Chi^2^ = 0.05, *p* = 0.8).

In the field plantation, IR*pmt* plants lost significantly more leaf area to herbivores than did WT plants (Figure 4B), demonstrating that nicotine indeed functions as a direct resistance trait of N. attenuata in its native habitat. Over a period of 16 d, IR*pmt* plants exposed to naturally occurring herbivores lost 16% of their total leaf area to herbivores, an amount that is more than double the amount of damage incurred by WT plants. In order to meet compliance requirements described in the Code of Federal Regulations (7CFR340.3c) for the introduction of organisms altered through genetic engineering, flowers were removed as they matured, and therefore we could not directly measure the fitness consequences of this greater herbivore load. However, in other experiments with N. attenuata plants grown in natural populations, leaf area damage is negatively correlated with capsule number (Baldwin 1998; Kessler and Baldwin 2004), suggesting that the strongly enhanced herbivore damage of the nicotine-deficient IR*pmt* plants translates into a fitness loss.

IR*pmt* plants were attacked by a variety of insect herbivores. About half of the total herbivore damage resulted from S. exigua feeding (Figure 4C). One-third of the total herbivore damage was damage from grasshoppers of the genus *Trimerotropis,* which followed the same general pattern of distribution as S. exigua damage, but the differences between unelicited IR*pmt* and WT plants were not significant. The damage caused by Epetrix hirtipennis was variable but significantly higher for unelicited IR*pmt* compared to WT plants (ANOVA: F = 2.81, df = 3, *p* = 0.04, *p*_PMT-WT_ < 0.05).

MeJA elicitation significantly reduced the damage of IR*pmt* plants to levels found on WT plants, suggesting that MeJA treatment elicits defense traits that are as efficient as the constitutive levels of nicotine in protecting plants. MeJA elicitation of N. attenuata plants is known to induce a diverse suite of transcriptional responses and secondary metabolites including TPIs, phenolics, flavonoids, phenolic putrescine conjugates, diterpene sugar esters, and volatile organic compounds (Halitschke and Baldwin 2003; Roda and Baldwin 2003), some of which apparently function as resistance traits. Which component of this complex suite of elicited metabolites is as effective as nicotine remains to be determined. It should be noted that the overall amounts of leaf area lost to herbivores was relatively low during the field experiments. Only 5% of the canopy area was lost from control and MeJA-elicited WT plants. In previous experiments (Baldwin 1998), fitness differences were observed between control and MeJA-elicited WT plants in populations that had lost approximately 40% of their canopy area to herbivores.

Altogether, these results provide direct evidence for the defensive value of nicotine. In a field trial, we established that a native tobacco, which produces large amounts of nicotine, is better defended against its natural herbivores than are nicotine-deficient transformants of the same genetic background. This is likely mediated by the reduction of herbivore performance and by the fact that these phytophagous insects prefer low-nicotine diets. In contrast to studies demonstrating genetic correlations between the production of secondary metabolites and herbivore resistance (Berenbaum et al. 1986; Shonle and Bergelson 2000), the resistance effects established in this study can be directly attributed to the altered traits. The fact that the silencing of one enzyme in the nicotine biosynthetic pathway redirects metabolite flux, resulting in the accumulation of an apparently less toxic alkaloid, anatabine, underscores the importance of characterizing single-gene transformants for secondary effects.

### Conclusion

Plant secondary metabolites are widely accepted as essential components of a plant's direct defenses against its natural enemies, but unambiguous proof has been lacking, mainly because of the difficulty of altering the expression of single traits in plants and testing the consequences of these manipulations under natural conditions. Transformation technology has provided biologists with the ability to manipulate and study the ecological consequences of single-gene manipulations. To date, the technology has largely been used for the heterologous expression of resistance genes (e.g., *Bacillus thuringiensis d-endotoxin*) in agricultural systems (see Tian et al. [2003] for an elegant exception), and therefore has provided little evidence for the defensive value of endogenously expressed traits against a plant's native herbivore community. The scientific value of transgenically silencing endogenous genes in native plants to understand the ecological function of particular genes has been undermined by the polarized attitudes towards the use of genetically modified organisms in agriculture. Transgenic down-regulation of nicotine demonstrates that N. attenuata is under relentless herbivore pressure. Disabling this resistance trait, even in a year of low herbivore abundance, results in a large increase in opportunistic herbivory and supports the conclusion that secondary metabolites play an important role in explaining why the earth is largely green (Hairston et al. 1960).

## Materials and Methods

### 

#### Plant material and transformation

N. attenuata Torr. ex Watson (synonymous with N. torreyana Nelson and Macbr.; Solanaceae) grown from field-collected seeds (Baldwin 1998) and inbred 11 or 14 generations were used for transformation and all experiments. Seed germination and the Agrobacterium tumifaciens (strain LBA 4404)–mediated transformation procedure are described in Krügel et al. (2002). In order to silence the expression of the two *N. attenuata pmt* genes, plants were transformed with pCAMPMT1 and pNATPMT1 vectors, which contain a gene fragment of *pmt1* (which has 95% identity to *pmt2*) in an antisense orientation, and pRESC5PMT, which contains the *pmt* gene fragment twice in an inverted orientation separated by intron 3 of the Flaveria trinervia gene *pyruvate orthophosphate dikinase (pdk)* (for vector construction and plasmids see Figure S5 and Protocol S1). T_1_ plants were screened for hygromycin resistance (*hygromycin phosphotransferase II* gene of the vector pCAMBIA-1301) and constitutive and induced nicotine accumulation; homozygosity was determined by resistance screening of the T_2_ plants. Two independently transformed homozygous IR*pmt* lines (108 and 145) were further characterized by Southern blot analysis and by the rescuing of the transformation vector from genomic DNA into Escherichia coli to identify copy number and insertion site of the T-DNA (see Figure S1 and Protocol S1).

#### PMT mRNA accumulation and secondary metabolites.

Transformed lines (108 and 145) and WT plants were grown in 1-l hydroponic vessels in a climate chamber as described in Hermsmeier et al. (2001), and 4-wk-old rosette-stage plants were treated (elicited) on the first two fully expanded (source) leaves with 150 μg of MeJA per plant applied in 20 μl of lanolin paste, or left untreated. Approximately 200 mg of young roots was harvested and frozen in liquid nitrogen 10 h after elicitation, and RNA was extracted with Tri Reagent (Sigma, Taufkirchen, Germany) according to the manufacturer's instructions (*n* = 3/line/treatment). PMT transcript accumulation was analyzed by real-time PCR (ABI PRISM 7000; Applied Biosystems, Darmstadt, Germany). cDNA was generated from 20 ng of RNA with MultiScribe reverse transcriptase (Applied Biosystems), and amplified using the qPCR core reagent kit (Eurogentec, Searing, Belgium) and a probe and primers that were gene-specific (for sequences see Figure S6). For analysis of secondary metabolites, leaves growing one node above the sink-source transition leaf and young root tissue were harvested 4 d after elicitation (*n* = 8–10/line/treatment). Samples were analyzed by HPLC for alkaloids, caffeoylputrescine, chlorogenic acid, and rutin (Keinänen et al. 2001; Halitschke and Baldwin 2003). A peak occurring in IR*pmt* alkaloid extracts but not in extracts of WT N. attenuata was collected and identified by nuclear magnetic resonance imaging as anatabine (for spectra and method, see Protocol S1).

To determine whether a NA oversupply was responsible for the formation of anatabine in the transformed lines, we supplied 4-wk-old plants with either unlabeled or D_4_-NA ethyl ester (1 mM) in their hydroponic solution 24 h after MeJA elicitation (*n* = 4/line/treatment). After 4 d, the treated leaf was harvested and extracted as above, but analyzed by LC/MS/MS to detect incorporation of the deuterium into nicotine and anatabine (for instrument conditions, see Protocol S1).

To examine the release of *cis*-α-bergamotene in the transformed lines compared to WT, volatiles from hydroponically grown plants (*n* = 3–5/line/treatment) enclosed in open-top volatile collection chambers were collected for an 8 h period starting 24 h after MeJA elicitation of the first two source leaves, and analyzed by GC/MS (Halitschke et al. 2000). TPI activity in the MeJA-treated leaf 3 d after elicitation was analyzed in plants (*n* = 5/line/treatment) by radial diffusion activity assay (van Dam et al. 2001).

#### *M. sexta* performance and feeding choice

In the glasshouse, 2-wk-old seedlings were planted individually into 2-l pots with potting soil (C 410; Stender, Schermbeck, Germany) at 26–28 °C under 16-h supplemental light from Philips Sun-T Agro 400- or 600-W Na lights. For analysis of performance, newly eclosed M. sexta larvae (North Carolina State University, Raleigh, North Carolina, United States) were placed on the first-stem leaf of 8-wk-old WT and IR*pmt* (108 and 145) plants and allowed to feed for 14 d. Larval mass was recorded at 8, 10, 12, and 14 d.

The first feeding choice of M. sexta was determined by placing newly eclosed larvae in the center of a 3-cm–diameter cup containing, on opposite sides, 1.5-cm^2^ WT and IR*pmt* (108) leaf pieces and recording the leaf on which larvae started feeding (*n* = 44).

#### Resistance of WT and IR*pmt* plants to herbivores in the natural habitat.

In a field plantation (15 m × 18 m; GPS: lat 37°08′45′′N, long 114°01′12′′) in N. attenuata's natural habitat in southwest Utah, transformed IR*pmt* (108) and WT plants were exposed to naturally occurring herbivores dispersing from adjacent populations. To allow for spatial heterogeneity, plants were transplanted in a paired design (with 0.3 m and 1.5 m between plants of a pair and between pairs, respectively) in which plants were matched for equal rosette diameters. Plants were grown in soil (Potting Mix; Miracle-Gro, Marysville, Ohio, United States) for 5 wk after germination (Krügel et al. 2002), and were transplanted into the field plot (10 columns by 15 lines) in their 3.8-l pots. Seven days after transplantation, 30 WT and IR*pmt* plants were elicited with 150 μg of MeJA per plant applied in 20 μl of lanolin paste to the two youngest rosette leaves. Starting 4 d after transplantation, each plant was examined for damage and insects (including predators and eggs) every other day for 14 d. Damage amount was estimated as a percentage of the total leaf area, and the characteristic damage caused by caterpillars, beetles, grasshoppers, myrids, and leafhoppers was noted separately. The most abundant herbivores observed in the field plantation during the release were S. exigua, *Trimerotropis* spp., *E. hirtipennis,* and *D. undecimpunctata. M. sexta* and M. quinquemaculata occurred in the season only rarely, and no eggs were laid in the plantation during the 14 d. As plants began to elongate and produce flowers, they were examined daily, and all flowers were removed before opening and anthesis to meet the performance standards in the Code of Federal Regulations (7CFR340.3c). Consequently, direct fitness measures were unobtainable in this experiment.

For analysis of alkaloids and TPIs under field conditions, leaf samples of WT and IR*pmt* plants in the plot (*n* = 6) were taken 7 wk after transplantation and frozen (dry ice). To determine if the herbivore phenotype of IR*pmt* plants observed in glasshouse-grown plants was retained in plants grown under natural light conditions, the M. sexta choice experiment was repeated. The first feeding choices of freshly eclosed M. sexta larvae (North Carolina State University) and of adults of field-collected D. undecimpunctata and Trichobarus mucorea (Chrysomelidae and Curculionidae) found on N. attenuata were determined as described above.

## Supporting Information

Figure S1 Copy Number of T-DNA in the Two Studied IR*pmt* Lines (A) Southern blot analysis of two independently transformed N. attenuata IR*pmt* lines (108 and 145) and WT plants. Genomic DNA (15 μg) from individual plants of the three genotypes and the plasmid used for transformation pRESC5PMT (4 ng) were digested with EcoRV and blotted onto nylon membranes (Winz and Baldwin 2001). The blot was hybridized with a PCR fragment of the *hygromycin phosphotransferase II* gene from pCAMBIA-1301, which is specific for the selective marker on the T-DNA and signifies one insertion in each of the two lines.
(B) Ethidium bromide staining of the DNA revealed an overload of the DNA of the IR*pmt* lines and therefore loading of the WT was controlled by stripping and rehybridization with a PMT probe, which clearly revealed the endogenous *pmt1* and *pmt 2* genes described (Winz and Baldwin 2001) (unpublished data).
(6.3 MB TIF).

Figure S2 Secondary Metabolite Levels in the Studied IR*pmt* Lines Inverted-repeat silencing of *pmt* did not change the levels of (A) anabasine, (B) caffeoylputrescine, (C) chlorogenic acid, and (D) rutin (mean ± standard error [SE]) in two independently transformed N. attenuata lines (108 and 145) compared to WT plants. Plants were harvested 4 d after receiving one of four treatments: untreated control (Con), wounding (W), wounding and regurgitate application (W+R), and application of 150 μg of MeJA per plant applied in a lanolin paste. Plants were treated at the first two fully expanded (source) leaves and wounding was performed by generating three rows of puncture wounds on each leaf side using a pattern wheel. Subsequently, 10 μl per leaf of either water or M. sexta regurgitate diluted 1:1 (v:v) was dispersed over the puncture wounds (*n* = 8–10).
(179 KB PPT).

Figure S3 Proteinase Inhibitor and Volatile Emission of the Studied IR*pmt* Lines Levels of (A) TPI and (B) *cis-*α-bergamotene emission (mean ± SE) in two independently transformed N. attenuata IR*pmt* lines (108 and 145) did not differ from WT plants 4 d (for TPI) and 10 h (for *cis-*α-bergamotene) after receiving one of four treatments (as described for S2): untreated control (Con), wounding (W), wounding with additional regurgitate application (W+R), and MeJA elicitation. IS, internal standard.
(73 KB PPT).

Figure S4 Growth Parameters Under Glasshouse and Field Conditions of the Studied IR*pmt* Lines N. attenuata plants transformed with an IR*pmt* construct (108 or 145) did not differ in (A) stalk length [*n*_PMT_ = 43, *n*_WT_ = 57, *n*_PMT*_ and *n*_WT*_ = 28] and (B) rosette diameter [*n* = 8] from WT grown under either field (A) or glasshouse (B) conditions. Plants in (A) were untreated or elicited (*) with MeJA 7 d after plants were transplanted into a field plot in a native habitat.
(98 KB PPT).

Figure S5 Transformation Vectors This figure shows plasmids used for the generation of N. attenuata lines with reduced levels of two PMTs due to posttranscriptional gene silencing. Both (A) pCAMPMT1 (10.7 kb) and (B) pNATPMT1 (9.7 kb) allow the synthesis of *pmt* antisense RNA. (C) pRESC5PMT (12.4 kb) was used for the synthesis of *pmt* RNA capable of forming an inverted repeat. Functional elements: *bla*, beta-lactamase gene from plasmid pUC19; *hptII*, gene for hygromycin resistance from pCAMBIA-1301; LB and RB, left and right border of T-DNA; *nptIII*, aminoglycoside phosphotransferase of type III from Streptococcus faecalis; ori ColE1, origin of replication from pUC19; ori pVS1, origin of replication from plasmid pVS1; P_CaMV_ and T_CaMV_, 35S promoter and terminator of cauliflower mosaic virus; *pdk i3*, intron 3 of *pdk; pmt1*, gene fragment of *pmt1* (95% identical with *N. attenuata pmt2*); P_NOS_ and T_NOS_, promoter and terminator of the *nopaline synthase* gene; *repA* pVS1, replication protein gene from pVS1; *sat-1*, nourseothricin resistance gene; *staA* pVS1, partitioning protein gene from pVS1. Displayed restriction sites mark the borders of functional elements, which are displayed in gray if on the T-DNA and in black if outside the T-DNA.
(56 KB PPT).

Figure S6 PMT Sequences and TaqMan Probe Nucleotide sequences of *N. attenuata pmt1* and *pmt2* mRNA (Winz and Baldwin 2001) aligned with ClustalW. Primers and probe (underlined) used for real-time PCR of *pmt* mRNA are highlighted and bold.
(396 KB TIF).

Protocol S1 Molecular and Analytical Methods (58 KB DOC).

### Accession Numbers

GenBank accession numbers for the genes discussed in this paper are *bla* from puc19 (L09137), *hygromycin phosphotransferase II* from pCAMBIA-1301 (AF234297), *pdk* (X79095), *pmt1* (AF280402), and *pmt2* (AF280403).

## Abbreviations

IR*pmt*: inverted-repeat *putrescine N-methyl transferase;* MeJA
NA: nicotinic acid
*pdk*: *pyruvate orthophosphate dikinase;* PMT
SE: standard error
T-DNA: transferred DNA
TPI: trypsin protease inhibitor
WT: wild-type
