# Correction: Nicotine's Defensive Function in Nature

**DOI:** 10.1371/journal.pbio.0020382

**Published:** 2004-10-12

## Abstract

July correction

**In *PLoS Biology*, volume 2, issue 8**

Nicotine's Defensive Function in Nature

**Anke Steppuhn, Klaus Gase, Bernd Krock, Rayko Halitschke, Ian T. Baldwin**

DOI: 10.1371/journal.pbio.0020217

The Academic Editor was erroneously listed as Michael Levine. The Academic Editor for this paper is Joy Bergelson, Chicago University.

This correction note may be found online at DOI: 10.1371/journal.pbio.0020382.

