# Supplementary figures and images for: Nicotine's Defensive Function in Nature
Source: PLoS Biol. 2004 Aug 17;2(8):e217. doi: 10.1371/journal.pbio.0020217 (PMC509292; doi:10.1371/journal.pbio.0020217)

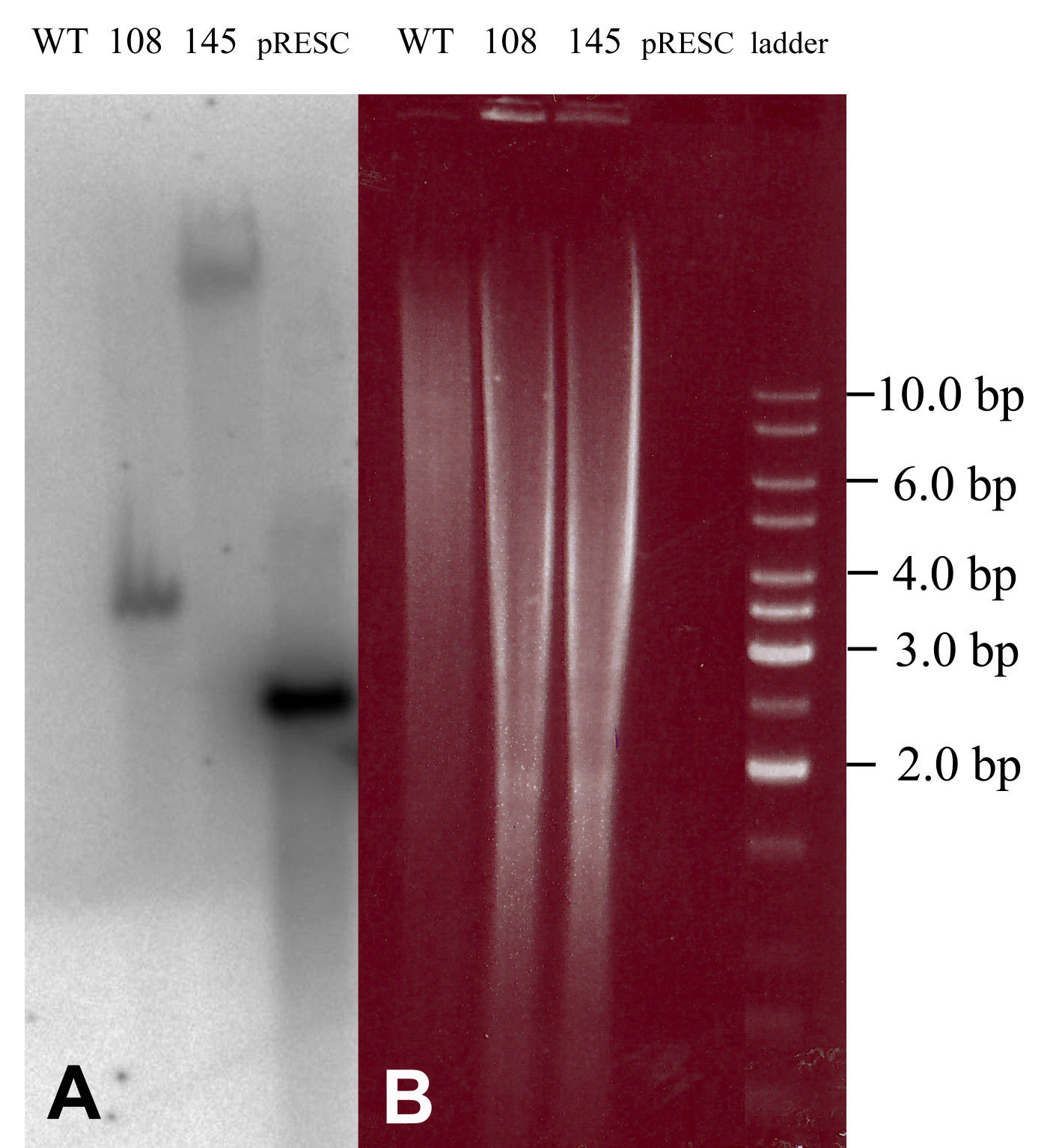

Supplement: Figure S1 — (A) Southern blot analysis of two independently transformed N. attenuata IRpmt lines (108 and 145) and WT plants. Genomic DNA (15 μg) from individual plants of the three genotypes and the plasmid used for transformation pRESC5PMT (4 ng) were digested with EcoRV and blotted onto nylon membranes (Winz and Baldwin 2001). The blot was hybridized with a PCR fragment of the hygromycin phosphotransferase II gene from pCAMBIA-1301, which is specific for the selective marker on the T-DNA and signifies one insertion in each of the two lines. (B) Ethidium bromide staining of the DNA revealed an overload of the DNA of the IRpmt lines and therefore loading of the WT was controlled by stripping and rehybridization with a PMT probe, which clearly revealed the endogenous pmt1 and pmt 2 genes described (Winz and Baldwin 2001) (unpublished data). (6.3 MB TIF). [file pbio.0020217.sg001.tif]

## Slide 1
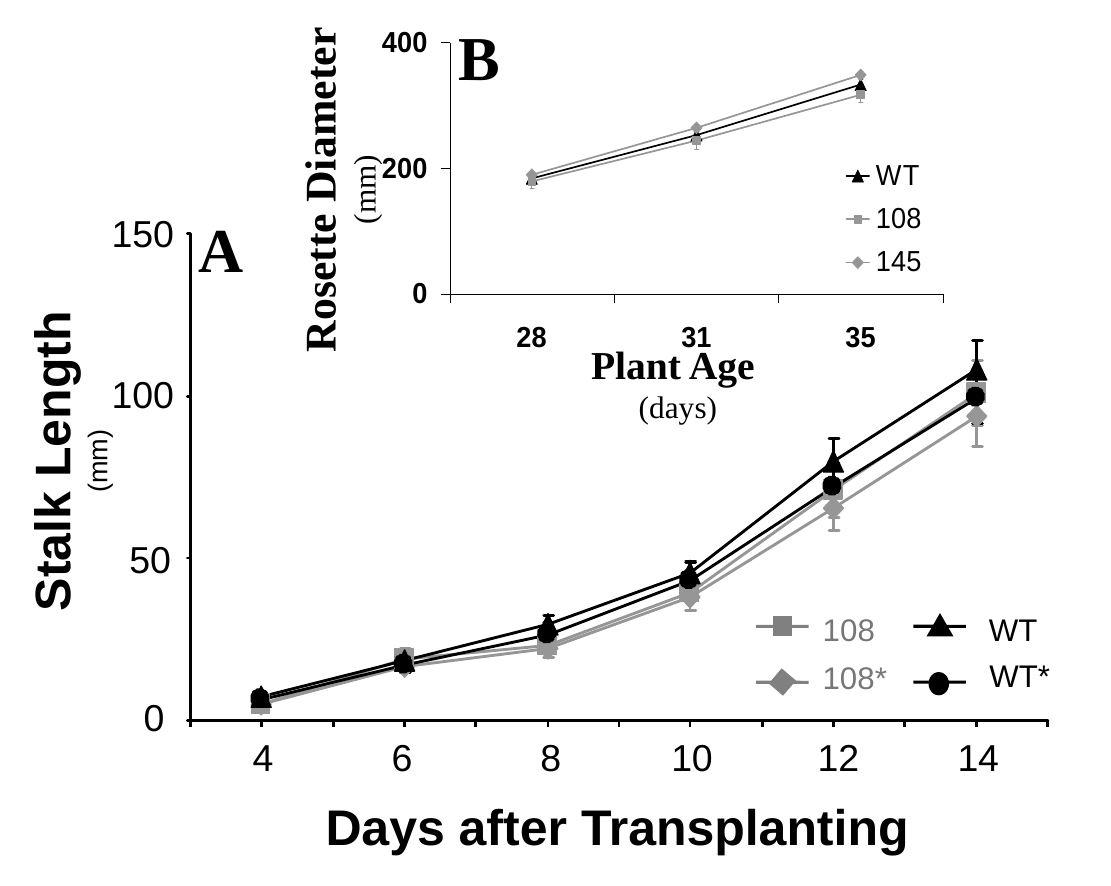

B
Rosette Diameter
(mm)
150
100
Stalk Length
(mm)
50
108
WT
WT*
108*
0
4
6
8
10
12
14
Days after Transplanting
A
Plant Age
(days)

Supplement: Figure S4 — N. attenuata plants transformed with an IRpmt construct (108 or 145) did not differ in (A) stalk length [n PMT = 43, n WT = 57, n PMT* and n WT* = 28] and (B) rosette diameter [n = 8] from WT grown under either field (A) or glasshouse (B) conditions. Plants in (A) were untreated or elicited (*) with MeJA 7 d after plants were transplanted into a field plot in a native habitat. (98 KB PPT). [file pbio.0020217.sg004.ppt]

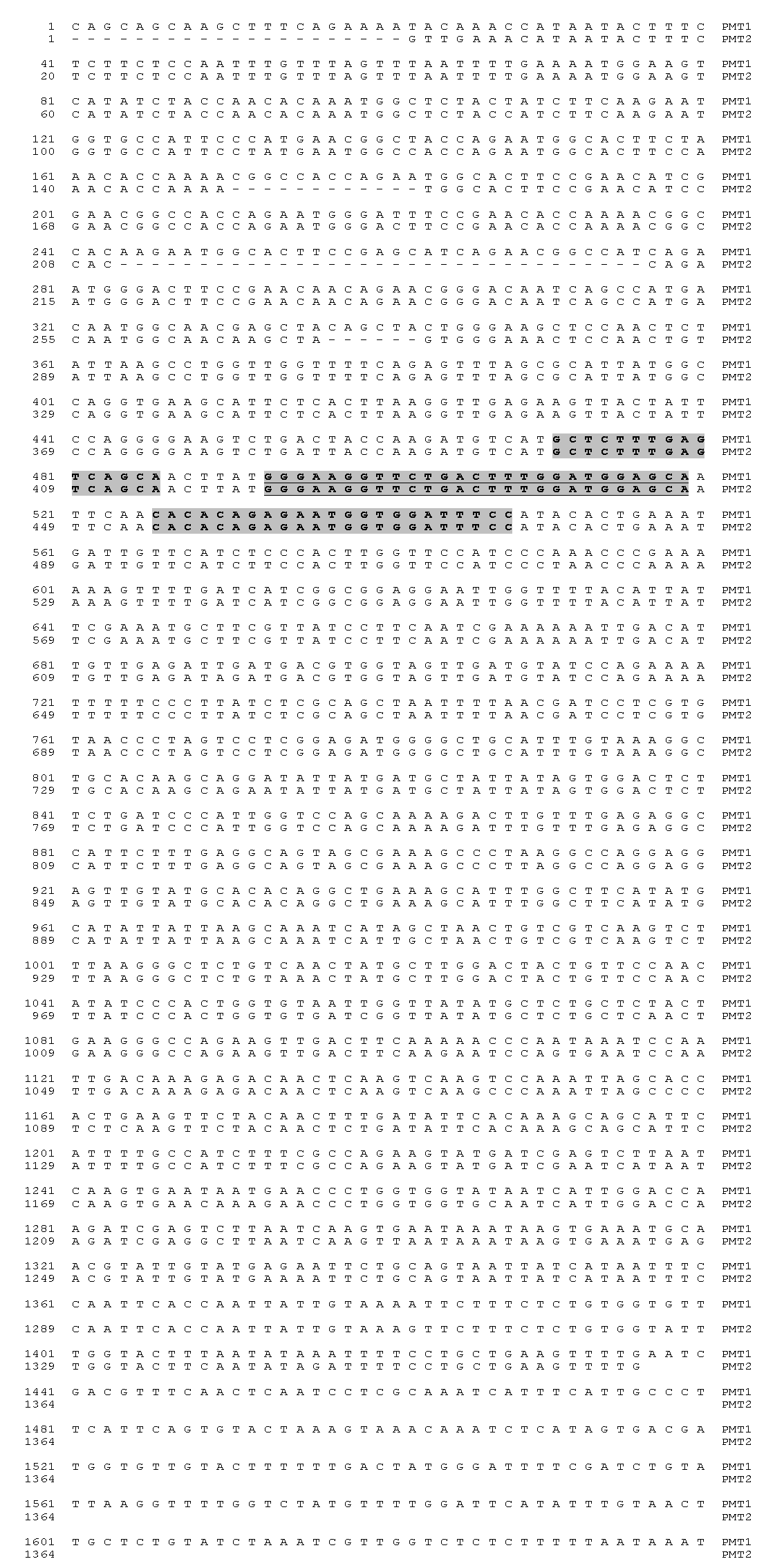

Supplement: Figure S6 — Nucleotide sequences of N. attenuata pmt1 and pmt2 mRNA (Winz and Baldwin 2001) aligned with ClustalW. Primers and probe (underlined) used for real-time PCR of pmt mRNA are highlighted and bold. (396 KB TIF). [file pbio.0020217.sg006.tif]
