# Supplementary material for: Nicotine's Defensive Function in Nature
Source: PLoS Biol. 2004 Aug 17;2(8):e217. doi: 10.1371/journal.pbio.0020217 (PMC509292; doi:10.1371/journal.pbio.0020217)
Supplement: Figure S2 — Inverted-repeat silencing of pmt did not change the levels of (A) anabasine, (B) caffeoylputrescine, (C) chlorogenic acid, and (D) rutin (mean ± standard error [SE]) in two independently transformed N. attenuata lines (108 and 145) compared to WT plants. Plants were harvested 4 d after receiving one of four treatments: untreated control (Con), wounding (W), wounding and regurgitate application (W+R), and application of 150 μg of MeJA per plant applied in a lanolin paste. Plants were treated at the first two fully expanded (source) leaves and wounding was performed by generating three rows of puncture wounds on each leaf side using a pattern wheel. Subsequently, 10 μl per leaf of either water or M. sexta regurgitate diluted 1:1 (v:v) was dispersed over the puncture wounds (n = 8–10). (179 KB PPT). [file pbio.0020217.sg002.ppt]

## Slide 1
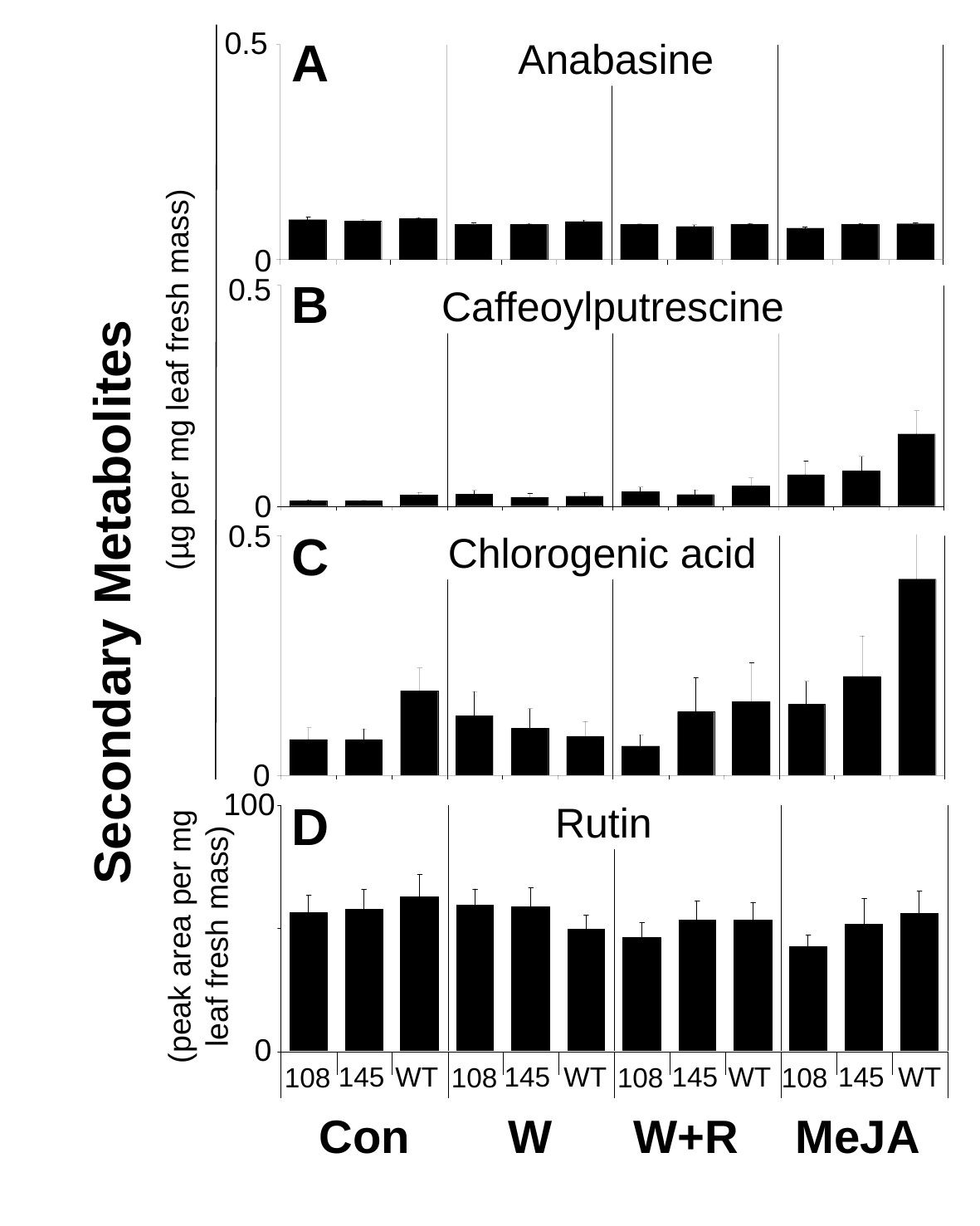

0.5
A
Anabasine
0
0.5
B
Caffeoylputrescine
(µg per mg leaf fresh mass)
0
0.5
C
Chlorogenic acid
Secondary Metabolites
0
100
D
Rutin
(peak area per mg leaf fresh mass)
0
145
WT
145
WT
145
WT
145
WT
108
108
108
108
Con
W
W+R
MeJA
