# Supplementary material for: Nicotine's Defensive Function in Nature
Source: PLoS Biol. 2004 Aug 17;2(8):e217. doi: 10.1371/journal.pbio.0020217 (PMC509292; doi:10.1371/journal.pbio.0020217)
Supplement: Figure S3 — Levels of (A) TPI and (B) cis-α-bergamotene emission (mean ± SE) in two independently transformed N. attenuata IRpmt lines (108 and 145) did not differ from WT plants 4 d (for TPI) and 10 h (for cis-α-bergamotene) after receiving one of four treatments (as described for S2): untreated control (Con), wounding (W), wounding with additional regurgitate application (W+R), and MeJA elicitation. IS, internal standard. (73 KB PPT). [file pbio.0020217.sg003.ppt]

## Slide 1
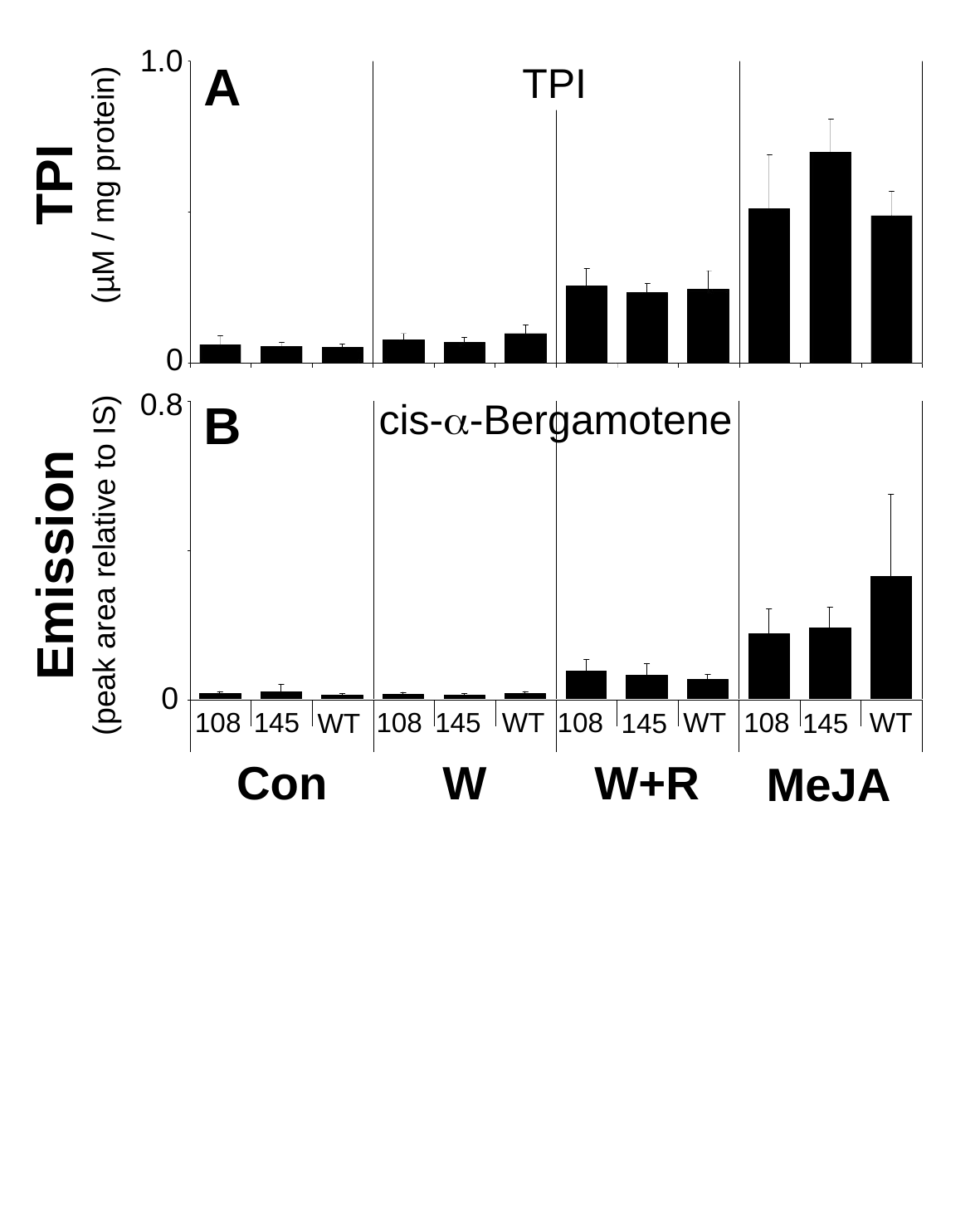

1.0
A
TPI
TPI
(µM / mg protein)
0
0.8
B
cis-α-Bergamotene
Emission
(peak area relative to IS)
0
108
145
108
145
WT
108
WT
108
WT
WT
145
145
Con
W
W+R
MeJA
