# Supplementary material for: Nicotine's Defensive Function in Nature
Source: PLoS Biol. 2004 Aug 17;2(8):e217. doi: 10.1371/journal.pbio.0020217 (PMC509292; doi:10.1371/journal.pbio.0020217)
Supplement: Figure S5 — This figure shows plasmids used for the generation of N. attenuata lines with reduced levels of two PMTs due to posttranscriptional gene silencing. Both (A) pCAMPMT1 (10.7 kb) and (B) pNATPMT1 (9.7 kb) allow the synthesis of pmt antisense RNA. (C) pRESC5PMT (12.4 kb) was used for the synthesis of pmt RNA capable of forming an inverted repeat. Functional elements: bla, beta-lactamase gene from plasmid pUC19; hptII, gene for hygromycin resistance from pCAMBIA-1301; LB and RB, left and right border of T-DNA; nptIII, aminoglycoside phosphotransferase of type III from Streptococcus faecalis; ori ColE1, origin of replication from pUC19; ori pVS1, origin of replication from plasmid pVS1; PCaMV and TCaMV, 35S promoter and terminator of cauliflower mosaic virus; pdk i3, intron 3 of pdk; pmt1, gene fragment of pmt1 (95% identical with N. attenuata pmt2); PNOS and TNOS, promoter and terminator of the nopaline synthase gene; repA pVS1, replication protein gene from pVS1; sat-1, nourseothricin resistance gene; staA pVS1, partitioning protein gene from pVS1. Displayed restriction sites mark the borders of functional elements, which are displayed in gray if on the T-DNA and in black if outside the T-DNA. (56 KB PPT). [file pbio.0020217.sg005.ppt]

## Slide 1
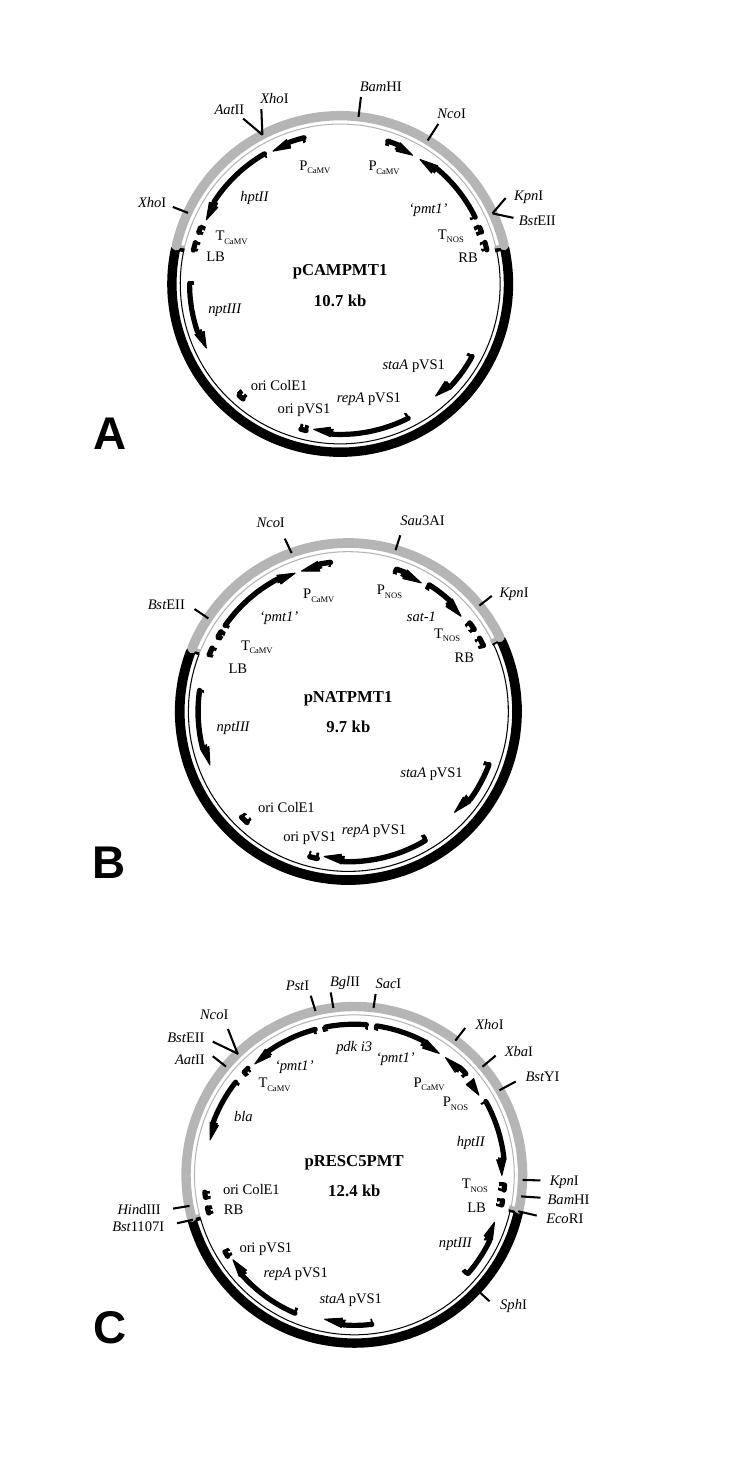

BamHI
XhoI
AatII
NcoI
PCaMV
PCaMV
KpnI
hptII
XhoI
‘pmt1’
BstEII
TNOS
TCaMV
LB
RB
pCAMPMT1
10.7 kb
nptIII
staA pVS1
ori ColE1
repA pVS1
ori pVS1
A
Sau3AI
NcoI
PNOS
KpnI
PCaMV
BstEII
‘pmt1’
sat-1
TNOS
TCaMV
RB
LB
pNATPMT1
9.7 kb
nptIII
staA pVS1
ori ColE1
repA pVS1
ori pVS1
B
BglII
SacI
PstI
NcoI
XhoI
BstEII
pdk i3
XbaI
‘pmt1’
AatII
‘pmt1’
BstYI
PCaMV
TCaMV
PNOS
bla
hptII
pRESC5PMT
12.4 kb
KpnI
TNOS
ori ColE1
BamHI
LB
HindIII
RB
EcoRI
Bst1107I
nptIII
ori pVS1
repA pVS1
staA pVS1
SphI
C
