# Supplementary material for: Nicotine's Defensive Function in Nature
Source: PLoS Biol. 2004 Aug 17;2(8):e217. doi: 10.1371/journal.pbio.0020217 (PMC509292; doi:10.1371/journal.pbio.0020217)
Supplement: Protocol S1 — (58 KB DOC). [file pbio.0020217.sd001.doc]

**Supporting Information: Protocol 1**

**I. Vector construction:** The construction of vectors pCAMPMT1 (10.7 kb; Fig. 9A) and pNATPMT1 (9.7 kb; Fig. 9B), both containing the same 0.9 kb cDNA portion of the *Nicotiana attenuata* putrescine N-methyltransferase 1 gene (*pmt1*; GenBank AF280402, Fig. 10) expressed in antisense orientation, were described elsewhere (Krügel et al. 2002), and (Hui et al. 2003), respectively).

In order to obtain a basic cloning vector with two inverted copies of a gene fragment separated by an intron functional in *Nicotiana attenuata*, cDNA of the *N. benthamiana* phytoene desaturase gene (*pds*; GenBank AJ571700) resident on a plasmid kindly provided by D. Baulcombe, JIC Norwich, United Kingdom, was PCR amplified using primer pairs PDS13-40 (5´-GCGGCGAAGCTTGAGCTCAGGCACTCAACTTTATAAACCC-3´), pDS14-40 (5´-GCGGCGGAATTCCTCGAGTCAGCTCGATCTTTTTTATTCG-3´) and PDS15-40 (5´-GCGGCGAAGCTTCTGCAGAGGCACTCAACTTTATAAACCC-3´), pDS16-40 (5´- GCGGCGGAATTCCCATGGTCAGCTCGATCTTTTTTATTCG-3´). After digestion with *Eco*RI and *Hin*dIII both 0.2 kb PCR fragments were cloned in pUC18 (Norrander et al. 1983; GenBank L08752) cut with the same enzymes, yielding pUCPDS4 (2.9 kb) and pUCPDS5 (2.9 kb), respectively. After treatment with *Pst*I, T4 DNA Polymerase and *Eco*RI the 4.5 kb fragment of pHYB34 (Gase et al. 1998), served as cloning vector for the 0.2 kb *pds* fragment obtained by treating pUCPDS4 with *Hin*dIII, T4 DNA Polymerase and *Eco*RI, resulting in pUCPDS6 (4.2 kb). pUCPDS7 (4.5 kb) was created by fusing the 2.4 kb *Aat*II-*Hin*dIII vector fragment of pUCPDS5 to the 2.1 kb *´pds´* containing fragment of pUCPDS6. To prepare an appropriate plant transformation vector the 9.8 kb *Bst*EII- *Nco*I fragment of pRESC20 (Zavala et al. 2004) was blunt ended with T4 DNA Polymerase and circularized. The resulting plasmid pRESC201 (9.8 kb), cut with *Nco*I and *Xho*I, served as cloning vector for the 1.8 kb *Nco*I-*Xho*I *´pds´* inverted repeat fragment from pUCPDS7, yielding pRESC500 (11.5 kb). Intron 3 of the *Flaveria trinervia* pyruvate, orthophosphate dikinase gene (GenBank X79095) resident on a plasmid kindly provided by P. Westhoff, Düsseldorf, Germany, was PCR amplified with primers PDK1-29 (5´-GCGGCGGAGCTCCTTGGTAAGGAAATAAT-3´) and PDK2-30 (5´-GCGGCGCTGCAGTCCCAACTGTAATCAATC-3´). After digestion with *Pst*I and *Sac*I the resulting 0.8 kb PCR fragment was used to replace the 1.4 kb *Pst*I- *Sac*I spacer between both *´pds´* copies on pRESC500, yielding pRESC501 (10.9 kb) which allowed efficient silencing of the *pds* gene in *N. attenuata* (data not shown), thus proving the function of the inverted repeat structure. The 0.2 kb *Nco*I-*Pst*I *pds* fragment of pRESC501 was replaced with a 0.9 kb *Nco*I-*Pst*I fragment containing the *pmt1* cDNA portion resident on pCAMPMT1, PCR synthesized with Primers PMT7-31 (5´-GCGGCGCCATGGAGCCCTTAAAGACTTGACG-3´) and PMT8-33 (5´-GCGGCGCTGCAGTACCAACACAAATGGCTCTAC-3´). In the resulting plasmid pRESC503 the 0.2 kb *Sac*I-*Xho*I *pds* fragment was replaced with a 0.9 kb *Sac*I-*Xho*I fragment carrying the *pmt1* cDNA portion of pCAMPMT1, PCR amplified with primers PMT9-33 (5´-GCGGCGCTCGAGCGAGCCCTTAAAGACTTGACG-3´) and PMT10-33 (5´-GCGGCGGAGCTCTACCAACACAAATGGCTCTAC-3´), thus yielding plant transformation vector pRESC5PMT (12.3 kb; Fig. 9C).

**II. Plasmid Rescuing:** We took advantage of the presence of the pUC19 (Yanischperron et al. 1985; GenBank L09137) origin of replication and the beta-lactamase gene as selectable marker, which are both functional in *E. coli,* to recover the plant transformation vector pRESC5PMT T-DNA integrated into the *N. attenuata* chromosome. This rescuing was performed as follows: chromosomal DNA of the two independently transformed homozygous *N. attenuata* lines, 108 and 145, was isolated from 2 week-old plants as described previously (Krügel et al. 2002). A mixture of 250 µl A1, 250 µl A2 and 300 µl A3 from the Macherey & Nagel (Düren, Germany) Nucleospin Kit was prepared and added to 50 µl of DNA solution. Subsequently the chromosomal DNA was purified with the kit as recommended for pDNA. Each 2 µg of the obtained DNA were cut with *Bcl*I, *Eco*RI or *Hin*dIII. After a precipitation step, the pellets were washed with 70% ethanol. Circularization of the fragments was done with T4 DNA ligase for 16 hr in a final volume of 14 µl. *E. coli* TOP10 (Grant et al. 1990; F- *mcr*A Δ(*mrr-hsd*RMS-*mcr*BC} φ80*lac*ZΔM15 Δ*lac*X74 *deo*R *rec*A1 *ara*D139 Δ (*ara-leu*)7697 *gal*U *gal*K *rps*L (Str^R^) *end*A1 *nup*G) was transformed with the circularized chromosomal DNA fragments. After selection on 100 µg/ml Ampicillin, 10 *E. coli* clones with rescued plasmids per digestion were isolated if rescuing was successful.

Nine rescued plasmids obtained from the different digestions were sequenced with primers binding close to and reading over the borders of pRESC5PMT T-DNA. This allowed us to identify the integration site of the T-DNA into the *N. attenuata* chromosome and to determine if the expected pRESC5PMT region was integrated. In line 108, the exact T-DNA fragment was fully integrated, whereas in line 145, a 227 bp deletion occurred: the integrated T-DNA comprised the region from right border to the middle of the NOS-terminator; the 3´ region of the NOS-terminator and the left border sequences were deleted. However, the integrated T-DNA is still functional, since the plants of this transformed line are hygromycin resistant and nicotine deficient. The analysis of the sequenced clones for line 108 revealed the T-DNA integration occurred into a gene homologous to GenBank CB214928 (SAM Demethylmenaquinone methyltransferase like gene from *Lycopersion esculentum*). The integration in Line 145 was identified to be in a region homologous to GenBank BI933931 (cDNA from the anthesis of *L. esculentum* flower).

**III. ^1^H-NMR of anatabine (D_4_-MeOH).** NMR measurement was performed on a Bruker DRX 500 NMR spectrometer, operating at 500,13 MHz for ^1^H. The sample was measured in D_4_-MeOH. Chemical shifts are given in δ values referring to TMS as internal standard and coupling constants in Hz. Literature data are given in square brackets (Yang and Tanner 1997: 2.62 (d) 3´H; 2.73 (m) 3´H [2.18, m]; 3.79 (d 16.0) 6´H [3.40, dm 17.0, 4.0]; 3.95 (d 16.0) 6´H [3.53 dm, 17.0, 2.0]; 4.59 (dd 11.6, 4.7) 2´H [3.80, t 7.0]; 5.90 (d 10.0) 4´H [5.78, d 10.0]; 6.10 (d 10.0) 5´H [5.72, ddd 10.0, 4.0, 2.0]; 7.60 5H [7.20, ddd 8.0, 5.0, 0.8]; 8.03 (d 8.0) 4H [7.67, dt 8.0, 1.8, 2.0]; 8.67 6H [8.42, dd 5.0, 1.8]; 8.72 2H [8.54, d 2.0]).

**IV. Analysis of nicotine and anatabine and their deuterated forms by LC/MS-MS.** The samples were measured by an Agilent LC1100 HPLC system (Agilent, Waldbronn, Germany) with degasser, binary pump, auto injector, column thermostat, and DAD coupled with a LCQ DECA XP mass spectrometer (Thermo-Finnigan, Egelsbach, Germany). Mobile phase A consisted of 0.5% NH_4_OH in water and mobile phase B of 0.5% NH_4_OH in acetonitrile. The mobile phase gradient was linearly increased from 20 % B (initial value) to 100 % B at 18 min. The mobile phase flow was 1.0 mL/min and the injection volume 15 µL. Stationary phase was a Luna 5u C18 column (250 x 4.60 mm, 5 μm particle size, Phenomenex, Aschaffenburg, Germany). Under these conditions anatabine eluted at 6.9 min and nicotine at 8.6 min. The MS conditions were as follows: APCI Ion source, positive polarity; 500°C vaporizer temperature; 175°C capillary temperature; 10 μA discharge current. 6 scan events (MS/MS experiments) were programmed: 1. m/z [161 @ 27](mailto:161@27) collision energy (arbitrary units) for anatabine; 2. m/z [163 @ 27](mailto:161@27) for nicotine and D_2_-anatabine; 3. m/z [165 @ 27](mailto:161@27) for D_2_-nicotine and D_4_-anatabine; 4. m/z [167 @ 27](mailto:161@27) for D_4_-nicotine and D_6_-anatabine; 5. m/z [169 @ 27](mailto:161@27) for D_6_-nicotine and D_8_-anatabine; 6. m/z [171 @ 27](mailto:161@27) for D_8_-nicotine.

**Literature cited**

Gase K, Ozegowski J, Malke H (1998) The *Streptococcus agalactiae* hylB gene encoding hyaluronate lyase: completion of the sequence and expression analysis. Biochim Biophys Acta-Gene Struct Expression 1398(1): 86-98.

Grant SGN, Jessee J, Bloom FR, Hanahan D (1990) Differential plasmid rescue from transgenic mouse DNAs into *Escherichia coli* methylation-restriction mutants. Proc Natl Acad Sci USA 87(12): 4645-4649.

Hui DQ, Iqbal J, Lehmann K, Gase K, Saluz HP et al. (2003) Molecular interactions between the specialist herbivore *Manduca sexta* (Lepidoptera, Sphingidae) and its natural host *Nicotiana attenuata*: V. Microarray analysis and further characterization of large-scale changes in herbivore-induced mRNAs. Plant Physiol 131(4): 1877-1893.

Krügel T, Lim M, Gase K, Halitschke R, Baldwin IT (2002) *Agrobacterium*-mediated transformation of *Nicotiana attenuta*, a model ecological expression system. Chemoecology 12: 177-183.

Norrander J, Kempe T, Messing J (1983) Construction of improved M13- vectors using oligodeoxynucleotide-directed mutagenesis. Gene 26(1): 101-106.

Winz RA, Baldwin IT (2001) Molecular interactions between the specialist herbivore *Manduca sexta* (Lepidoptera, Sphingidae) and its natural host *Nicotiana attenuata*. IV. Insect-induced ethylene reduces jasmonate-induced nicotine accumulation by regulating putrescine N-methyltransferase transcripts. Plant Physiol 125(4): 2189-2202.

Yang CM, Tanner DD (1997) A simple synthesis of (+/-)-1,2,3,6-tetrahydro-2,3'-bipyridine (anatabine) and (+/-)-3-(2-piperidinyl)pyridine (anabasine) from lithium aluminum hydride and pyridine. Can J Chem-Rev Can Chim 75(6): 616-620.

Yanischperron C, Vieira J, Messing J (1985) Improved M13 phage cloning vectors and host strains - nucleotide- sequences of the M13mp18 and Puc19 vectors. Gene 33(1): 103-119.

Zavala JA, Patankar AG, Gase K, Baldwin IT (2004) Constitutive and inducible trypsin proteinase inhibitor production incurs large fitness costs in *Nicotiana attenuata*. Proc Natl Acad Sci USA 134(3): 1181-1190.
